# Early risk factors for anxiety disorders in children with autism spectrum disorders: results from the ELENA Cohort

**DOI:** 10.1038/s41598-022-15165-y

**Published:** 2022-06-28

**Authors:** Florine Dellapiazza, Cécile Michelon, Marie-Christine Picot, Amaria Baghdadli

**Affiliations:** 1 Centre de Ressources Autisme Languedoc-Roussillon et Centre d’Excellence sur l’Autisme et les Troubles Neuro-développementaux, CHU Montpellier, 39 Avenue Charles Flahaut, 34295 Montpellier cedex 05, France; 2 Clinical Research and Epidemiology Unit, Department of Medical Information, University Hospital, CHU Montpellier, Montpellier, France; 3 Université Paris-Saclay, UVSQ, Inserm, CESP, Team DevPsy, 94807 Villejuif, France; 4 Faculté de Médecine, Université de Montpellier, Montpellier, France

**Keywords:** Psychiatric disorders, Paediatric research

## Abstract

Anxiety in children with autism spectrum disorder (ASD) negatively affects their social interactions, and quality of life. It is necessary to identify early risk factors for anxiety to tailor prevention and interventions. We aimed to examine the clinical level of anxiety in children with ASD from 5 to 10 years of age and identify potential early risk factors 3 years earlier. Participants were ASD children included in ELENA, a French prospective cohort. In this study, we used the collection of data at Time 1-T1 (at baseline) and Time 2-T2 (3 years after T1). Two groups were identified at T2 according to the threshold for anxiety on the CBCL: ASD-only group and ASD + anxiety group. Our results showed that half of the children in our sample had a clinical level of anxiety at T2. Regression analysis showed that greater ASD severity and lower sensory processing difficulties predicted lower anxiety, whereas higher levels of restricted and repetitive behaviours tended to predict higher levels of anxiety. The high prevalence of clinical-level anxiety in our sample suggests the need for specific assessment and targeted treatment of anxiety on a routine basis.

## Introduction

Autism spectrum disorder (ASD) is a frequently occurring condition observed in 1–2% of children^1^, clinically defined by the association of disturbances in social communication and restricted and repetitive behaviours (RRB)^2^. ASD symptoms emerge in early childhood and persist throughout life, with various outcome trajectories^3,4^.

Anxiety disorder, defined as excessive fear and worry that is difficult to control, associated with physical symptoms that cause distress and or functional impairment^2^, is frequently reported in children with ASD. A meta-analysis found that 40% of children with ASD had anxiety disorder during middle childhood, which was more frequent than for children with typical development (large effect size difference) or developmental delay (small effect size difference)^5,6^. Anxiety in children with ASD negatively affects their adaptive skills, social interactions and schooling^6–8^. Moreover, a recent study showed that a high level of anxiety in children with ASD contributed to reducing both the quality of life of the child and his/her parents^9^.

Available longitudinal studies have highlighted several potential risk factors for anxiety disorders in children with ASD. A meta-analysis of 83 studies and two longitudinal studies found that children with a higher intelligence quotient (IQ) had a greater risk of having a higher level of anxiety^5,6,10^. Indeed, the meta-analysis showed that higher IQ and adaptive skills in toddlers were associated with higher levels of generalized anxiety symptoms in adolescence^10^. In their long-term longitudinal study, Gotham et al. found that verbal IQ positively correlated with anxiety symptoms^5^. Moreover, their study showed that the most predictive factor of anxiety among children with ASD was gender, girls being at a higher risk of having anxiety^5^. Two studies reported that higher levels of RRB in toddlerhood predicted higher levels of anxiety and could be an early risk factor of anxiety symptoms^10–12^. Conversely, Teh et al. showed that RRB was not a significant risk factor when the level of anxiety at baseline was controlled^13^. However, the heterogeneity of the chronological age of the sample (from 5 to 17 years at baseline) may have influenced their results. Another study also did not find an association between RRB and anxiety^14^. Two studies showed that anxiety symptoms contributed to higher levels of social communication impairment over time^14,15^. Another research showed that atypical sensory processing emerged earlier than anxiety in development, and could predict later development of anxiety^16^.

Data on the contribution of parental characteristics to anxiety in children with ASD is conflicting. Gotham et al. found that lower maternal education was associated with a higher level of internalizing behaviours in ASD^5^. Another study showed maternal stress to be associated with anxiety in children with ASD, but did not collected data concerning paternal stress^17^. In 2017, a meta-analysis^6^ had already highlighted the paucity of longitudinal studies on the impact of parent and child characteristics on anxiety in children with ASD that could help our understanding of anxiety factors in ASD and this scarcity of literature persists.

Finally, data from longitudinal studies about the early child and family characteristics that contribute to the development of anxiety in ASD are limited and conflicting. Further study of early risk factors for anxiety is thus essential to improve the prevention and treatment of anxiety disorder in children with ASD^18^.

We aimed to examine the clinical level of anxiety in children with ASD from 5 to 10 years of age (T2) and identify potential early risk factors 3 years earlier (T1). According to findings from previous longitudinal studies, we hypothesized that high levels of RRB or atypical sensory processing and a high IQ would be early risk factors of anxiety disorder in children with ASD.

## Results

### Participants

Overall, there were 114 participants, mostly males (N = 94; 82.5%), with a mean age of 4.6 years (N = 114; SD = 1.6) at T1 and 7.6 years (N = 114; SD = 1.6) at T2. The mean duration of follow-up of participants between T1 and T2 was 2.9 years (SD = 0.4). Their clinical characteristics are presented in Table 1.

**Table 1:** Clinical characteristics of the sample at T1 and T2 (N = 114).

	T1	T2
	Mean ± SD (N)	Mean ± SD (N)
Age	4.6 ± 1.6 (114)	7.6 ± 1.6 (114)
% (N) Male	82.5 (114)	82.5 (114)
ADOS-CSS	6.9 ± 1.8 (103)	7.5 ± 1.7 (106)
IQ	69.8 ± 24.5 (101)	77.0 ± 29.1 (104)
ADI*		
Social interaction	15.7 ± 6.1 (104)	–
RRB	4.8 ± 2.4 (104)	–
**CBCL**		
Anxiety score standard	62.5 ± 9.8 (89)	62.7 ± 9.0 (114)
**VABS-II score**		
Communication	69.8 ± 15.3 (113)	70.3 ± 18.2 (114)
Socialization	71.3 ± 10.2 (113)	68.0 ± 15.1 (114)
Daily living skills	75.1 ± 12.4 (113)	70.7 ± 15.2 (114)
**ABC score**		
Irritability	36.0 ± 18.9 (93)	32.0 ± 22.3 (108)
Lethargy	26.4 ± 16.6 (92)	22.1 ± 18.7 (108)
Stereotypy	28.2 ± 20.1 (92)	28.9 ± 22.1 (107)
Hyperactivity	44.8 ± 22.9 (92)	41.1 ± 23.1 (109)
Total sensory profile	137.3 ± 20.0 (82)	131.2 ± 23.6 (85)
% (N) School attendance	89.7 (87)	96.8 (95)
**Parental SES**		
% (N) Low	34.7 (34)	43.1 (41)
% (N) Middle	31.6 (31)	26.3 (25)
% (N) High	33.7 (33)	30.5 (29)
**HADS mother anxiety**		
% (N) Low	25.7 (18)	46.4 (26)
% (N) Middle	30.0 (21)	23.2 (13)
% (N) High	44.2 (31)	30.4 (17)
**HADS father anxiety**		
% (N) Low	43.9 (25)	65.9 (27)
% (N) Middle	35.1 (20)	14.6 (6)
% (N) High	21. 0 (17)	19.5 (8)

*Assessed once during the development.

*SD* standard deviation, *ADOS CSS* autism diagnostic observation schedule calibrate severity scale, *CBCL* Child Behavior Checklist, *VABS-II* Vineland second version, *ABC* Aberrant Behaviors Checklist, *ADI-R* autism diagnostic interview-revised, *RRB* restrictive repetitive behaviour, *SES* socioeconomic status, *HADS* The hospital anxiety and depression scale.

### Prevalence of clinical level of anxiety in children with ASD during the 3 years of follow up

At T1 (N = 89), 45% (N = 40) of the sample showed a clinical level of anxiety by the Child Behavior Checklist (CBCL). At T2 (N = 114), 50% (N = 57) of the sample showed a clinical level of anxiety. Standard mean scores of the CBCL were reported in Table 1. We found significant differences in the anxiety score among participants for whom the CBCL was completed at T1 and T2 (N = 89) (X^2^, p < 10^–5^). Indeed, among children scoring above the clinical cut-off for anxiety at T1, the level of anxiety at T2 remained stable for 33 children (82.5%), and decreased to under the cut off for seven (17.5%). By contrast, among children scoring under the clinical cut off of anxiety at T1, the level of anxiety at T2 remained stable for 34 (69.4%) and increased above the cut off for 15 (30.6%).

### Potential early indicators at T1 of clinical anxiety in children with ASD at T2

Intergroup comparisons (ASD-only group vs. ASD + anxiety group) of the clinical characteristics at T1 by univariate analysis are presented in Table 2. At T1, intergroup comparisons showed that individuals in the ASD + anxiety group consisted of mainly boys (X^2^_(1)_ = 3.8, p = 0.048, Cohen's w = 3.8), were older (Z = − 2.42, p = 0.01, Cohen's d = 0.53), showed lower ASD severity by the Autism Diagnostic Observation Schedule Calibrate Severity Score (ADOS-CSS) (Z = 3.18, p = 0.001, Cohen's d = 0.67), and had higher IQs (t = − 2.01, p = 0.021, Cohen's d = 0.47) than individuals in the ASD-only group. Moreover, at T1, individuals in the ASD + anxiety group showed more challenging behaviors, such as stereotypy (Z = − 2.31, p = 0.02, Cohen's d = 0.5), hyperactivity (t = − 2.70, p = 0.008, Cohen's d = 0.6), and irritability (t = − 4.19, p = 0.0001, Cohen's d = 0.87), a higher level of RRB (Z = − 2.18, p = 0.03, Cohen's d = 0.57), and more difficulties with sensory processing (t = 3.77, p = 0.0001, Cohen's d = 0.8). Intergroup comparisons showed that maternal stress tended to be higher (X^2^_(2)_ = 5.5, p = 0.06, Cohen's w = 0.53) in the ASD + anxiety group than individuals in the ASD-only group. However, the two groups were similar in terms of the fathers’ anxiety level, parental SES, socialization, Autism Diagnostic Interview-Revised (ADI-R) severity, adaptive functioning, and the level of lethargy (all p > 0.05).

**Table 2:** Intergroup comparisons of clinical characteristics at T1 (N = 114).

Variables at T1	ASD-only	ASD + anxiety	P value	Effect size
	Mean ± SD (N)	Mean ± SD (N)		
Age	4.2 ± 1.4 (57)	5.0 ± 1.6 (57)	0.01^µ^	0.53*
% (N) male	75.4 (43)	89.5 (51)	0.048^α^	0.32**
ADOS- CSS	7.5 ± 1.8 (51)	6.3 ± 1.8 (52)	0.001^µ^	0.67*
IQ	63.7 ± 21.7 (50)	75.2 ± 26.4 (45)	0.021^β^	0.47*
**CBCL**				
Anxiety score standard	56.5 ± 7.0 (41)	67.7 ± 8.8 (48)	0.000^µ^	1.4*
**ADI**				
Social interaction	15.7 ± 6.2 (52)	15.6 ± 6.1 (52)	0.9^β^	0.02*
RRB	4.1 ± 1.8 (52)	5.4 ± 2.7 (52)	0.03^µ^	0.57*
**VABS-II score**				
Communication	67.1 ± 14.9 (57)	72.5 ± 15.3 (56)	0.08^µ^	0.36*
Socialization	70.9 ± 10.8 (57)	71.7 ± 9.6 (56)	0.68^β^	0.08*
Daily living skills	75.3 ± 12.5 (57)	74.9 ± 12.4 (56)	0.8^β^	0.03*
**ABC score**				
Irritability	27.6 ± 16.3 (41)	42.7 ± 18.4 (52)	0.000^β^	0.87*
Lethargy	23.3 ± 13.5 (41)	28.8 ± 18.4 (51)	0.11^β^	0.34*
Stereotypy	22.8 ± 18.6 (41)	32.6 ± 20.5 (51)	0.02^µ^	0.5*
Hyperactivity	37.8 ± 20.3 (41)	50.4 ± 23.6 (51)	0.008^β^	0.6*
Total sensory profile	145.8 ± 21.1 (37)	130.3 ± 16.0 (45)	0.000^β^	0.8*
**Parental SES**				
% (N) Low	37.0 (15)	34.6 (18)	0.9^α^	0.06**
% (N) Middle	30.4 (14)	32.7 (17)		
% (N) High	32.6 (17)	32.7 (17)		
**HADS mother anxiety**				
% (N) Low	38.7 (12)	15.4 (6)	0.06^α^	0.53**
% (N) Moderate	29.0 (9)	30.8 (12)		
% (N) High	32.3 (10)	53.8 (21)		
**HADS father anxiety**				
% (N) Low	51.9 (14)	36.7 (11)	0.4^α^	0.4**
% (N) Moderate	25.9 (7)	43.3 (13)		
% (N) High	22.2 (6)	20.0 (6)		

^α^X^2^ test; ^β^Student *t* test; ^µ^Mann–Whitney test;*Cohen’s d ≤ 0.2 small effect size, d = 0.5 moderate effect size and d ≥ 0.80 large effect size; **Cohen’s w ≤ 0.1 small effect size, w = 0.3 moderate effect size, w ≥ 0.5 large effect size.

*SD* standard deviation, *ADOS-CSS* autism diagnostic observation schedule calibrate severity scale, *CBCL* Child Behavior Checklist, *VABS-II* Vineland second version, *ABC* Aberrant Behaviors Checklist, *ADI-R* autism diagnostic interview-revised, *RRB* restrictive repetitive behaviour, *SES* socioeconomic status, *HADS* The hospital anxiety and depression scale.

For multivariate analysis, logistic regression analysis was performed. The results are presented in Table 3. Higher ADOS-CSS scores and higher Total Sensory Profile scores were associated with lower odds of having a clinical level of anxiety at T2 (OR = 0.38, 95% CI: [0.17; 0.8], p < 0.0175 and OR = 0.9, 95% CI: [0.87; 0.99], p = 0.035, respectively) after adjustment for anxiety level and IQ at T1. In addition, we found that higher RRB scores (ADI) at T1 tended to be significantly associated with a higher risk of a clinical level of anxiety at T2 (OR = 1.38, 95% CI: [0.97; 1.95], p = 0.07). Thus, more severe ASD symptoms (ADOS-CSS score) and lower sensory processing disorders at T1 predicted less anxiety at T2, whereas higher levels of RRB at T1 tended to predict a clinical level of anxiety at T2. The percentage of concordance between the observed and predicted values was 91.6% for this model and the Hosmer and Lemeshow test p value was 0.5, showing the model to be highly predictive. In sensitivity analysis, risk factors for anxiety at T2 were explored by multiple imputation of missing data and no significant difference were found for level of RRB and ADOS. However, sensory processing level were not significant after imputation of missing data.

**Table 3:** Multivariables logistic regression models.

Variable	Complete case logistic regression (case = 30/controls = 27)		Logistic regression with multiple imputation (case = 57/control = 57)	
	aOR of anxiety (95% CI)	p-value	aOR of anxiety (95% CI)	p-value
ADOS-CSS score	0.38 (0.17; 0.8)	0.0175	0.67 (0.5; 0.92)	0.01
Total sensory profile	0.9 (0.87; 0.99)	0.035	0.98 (0.94; 1.0)	0.1
RRB (ADI)	1.38 (0.97; 1.95)	0.07	1.2 (0.95; 1.4)	0.1

*Adjusted on anxiety level and IQ at T1.

*ADOS-CSS* autism diagnostic observation schedule calibrate severity scale, *RRB* restrictive repetitive behaviour.

## Discussion

Our results, based on the CBCL parental questionnaire, showed that 45% of the sample had a clinical level of anxiety at T1 and this prevalence increased to 50% at T2. Moreover, the clinical cut-off for anxiety remained stable for 82.5% of children between T1 and T2. The prevalence of anxiety disorder was higher than the 40% reported by a meta-analysis^6^. The persistence of anxiety disorder with age has already been noted in a previous 19-month follow-up study of children with ASD aged between 5 and 17 years^13^. The observation that there is both a strong prevalence of anxiety disorder in children with ASD and that it is stable with age corroborates the hypothesis of a developmental vulnerability to anxiety disorder in children^6^ with ASD. Such vulnerability, coupled with the known negative impact of anxiety disorder on adaptive functioning and the quality of life of children with autism^6,8,19^, makes it essential to explore this psychiatric condition in prospective studies.

Our study suggests that a high level of RRB tend to be considered as an early predictor of anxiety disorder for children with ASD. This has already been reported by a previous study, which also showed that although the level of RRB predicted anxiety disorder, the reverse was not true^11^. Another study showed that intolerance to change and cognitive rigidity were particularly associated with anxiety disorder^20^. A review indicated that stereotypies were early signs of anxiety disorder in young children with ASD^21^. Our results also showed that lower sensory processing difficulties predicted lower anxiety. In this sense, it has also been shown that a high level of sensory problems in early childhood for individuals with ASD predicts the occurrence of anxiety disorder at later stages. Indeed, hyper-reactivity to sensory stimuli in the environment is likely to contribute to anxiety disorder in children with ASD^22^. Some authors have postulated that sensory hyper-reactivity is linked to the occurrence of specific phobias^23^. One study suggested that sensory over-responsiveness was a precursor of anxiety disorder and an early developmental marker of it^16^. The authors of a review of the literature have argued that the association between sensory processing and anxiety disorder should be investigated further^23^ and the significant trend in our results also underlined the need for further investigations.

We observed that higher IQ was a risk factor for later anxiety disorder and that higher ASD symptom severity was protective. Previous studies have also reported that anxiety disorder tended to be more common among individuals with high functioning ASD^6,24^. One study noted that among children with ASD + anxiety, those with the highest IQ more often had generalized anxiety, whereas those with the lowest IQ more often had symptoms of separation anxiety^10^. However, these results must be interpreted with caution because anxiety symptoms may be under-identified in children with ASD, in particular among those who have the lowest IQ or the highest ASD symptom severity. Such caution is all the more necessary as most studies used self-administered questionnaires, which are easier to complete by individuals with high-functioning ASD. It is also known that the clinical presentation of anxiety disorder is different in ASD than in typical development, particularly because of the communication disturbances and challenging behaviours in ASD, leading to underestimation of its signs and its under-diagnosis^19,25^. Nonetheless, our findings of an elevated prevalence of anxiety disorder in children with ASD corroborate previous results reported by studies also using parent self-reported questionnaires and suggest that parental reports are useful for the identification of anxiety disorder in children with ASD in clinical practice^26^.

One of our interesting results was that mothers of children with ASD + anxiety disorder tended to be more anxious than mothers of children with ASD-only, which could be the expression of a genetic/familial vulnerability to anxiety^6^. On the other hand, mothers with anxiety disorder themselves may be more aware of its manifestation in their child and better able to identify it through self-questionnaires^27^. Of note, this association was not found for the fathers in our sample, which may be related to gender differences in coping strategies for dealing with the child’s anxiety disorder, with mothers using emotional strategies and fathers problem solving, which is more effective^28^. Interestingly, the anxiety disorder found in the children was not associated with their parents’ socio-economic status. Conversely, Gotham et al. found that a lower educational level of mothers was associated with more internalized behaviours in their children^5^. The paucity and heterogeneity of the findings in the literature on the association between parents’ socio-economic status and anxiety disorder in their children with ASD also merit further study^6^.

One of the strengths of our study was that it used data from a prospective cohort involving a large number of children with a confirmed diagnosis of ASD and that it includes a standardized clinical record of anxiety.

This study must be interpreted in light of certain limitations. First, although the sample size was quite large, missing data limited the multivariate analysis. However, this potential limitation can be mitigated by the stability of multiple imputations of the missing data. Second, although anxiety disorder was assessed using the CBCL, which is a parent questionnaire considered to be effective in detecting anxiety in children with ASD, the CBCL was originally designed to detect “typical anxiety"^26^ in the general population and may not be able to identify its atypical manifestations in ASD. Third, medication was not considered in analysis.

Given the high prevalence of anxiety disorder in ASD, this disorder should therefore be considered in the clinical assessment process and intervention plan for children with ASD. In addition, we found that certain risk factors for anxiety can be identified in early childhood at the time of diagnosis. Indeed, high levels of sensory difficulties and RRB at diagnosis appear to predict the development of anxiety disorder 3 years later, suggesting that this subgroup requires early and specific intervention. These signs could thus not only be considered as warning signals of the risk of anxiety disorder but also prompt the implementation of preventive and treatment measures. Future studies should examine whether early behavioural and cognitive intervention typically targeting core ASD symptoms (such as the Early Start Denver Model^29^ or Preschool Autism Communication Trial intervention and sensory integration therapy^22^) also reduces sensory difficulties and RBB and thus comorbid anxiety disorder in ASD.

Moreover, research on the prevention and treatment of anxiety disorder in ASD needs to be developed. Importantly, two meta-analyses have shown the positive effects of cognitive behavioural therapy on reducing anxiety in youths with high-functioning ASD, suggesting a way forward^24,30^. Other more ecological and routinely feasible approaches are yet to be defined. Indeed, a recent meta-analysis^31^ showed the positive effects of school programs based on cognitive behavioural therapy and mindfulness to reduce anxiety in typically developing children, the effects of which could also be investigated in ASD in view of the challenges of their schooling and better inclusion.

To conclude, our study highlights the high prevalence of anxiety disorder in children with ASD and these result suggests that specific assessment coupled with targeted treatment of anxiety disorder are needed on a routine basis. Consistent with previous studies, we found that high levels of sensory processing difficulties and RRB in early childhood were potential risk factors for later anxiety disorder in children with ASD. These findings underscore the need to tailor early anxiety disorder prevention and treatment measures in ASD. Further studies are needed to evaluate the effectiveness of early, specific treatment of anxiety symptoms for children with ASD.

## Methods

### Participants

Participants were recruited from a large cohort of children diagnosed with ASD, the ELENA cohort (Longitudinal Study of Children with Autism)^32^. They have a diagnosis of ASD clinically confirmed by a multidisciplinary team using a standardized process, including the Autism Diagnostic Observation Schedule 2 (ADOS-2)^33^ and the Autism Diagnostic Interview-Revised (ADI-R)^34^, administered by licensed and trained psychologists, a parental interview about the child’s adaptive functioning using the Vineland–II (VABS-II), and direct psychological examinations to assess IQ.

For the current study inclusion criteria were: (1) to have been followed for at least 3 years in the ELENA cohort, (2) to be aged from 5 to 10 years and 11 months at T2, and (3) to have parents who completed the CBCL. The only exclusion criteria were genetic disorders in accordance with previous literature on anxiety in ASD^10,11,14,35^.

### Ethics approval and consent to participate

The study and informed consent procedure were approved by the Ethics Committee on the Research of Human Subjects at Marseille Mediterranean and the National Commission for Computing and Liberties (CNIL. number DR-2015-393). All participating families signed an informed consent form. All methods were performed in accordance with the relevant guidelines and regulations.

### Material

All measures except the ADI-R were completed at T1 and T2.

### The Child Behavior Checklist (CBCL)^36^

Anxiety problems were assessed using the CBCL, a standardized caregiver-report that explores emotional and behavioral problems in children and adolescents aged from 1.5 to 18 years. The CBCL provides quantified risk scores for childhood mental health conditions. T-scores based on age and sex were obtained. The anxiety disorder subscale, called the DSM-oriented scale, allows the detection of anxiety disorder through 6 items. Raw scale scores are transformed to t-scores (M = 50; SD = 10) to allow for comparison with children of the same age and gender. T-scores ≥ 70 are considered clinically significant and T-scores from 65 to 69 borderline clinically significant. Internal consistency ranged from 0.72 to 0.91, inter-rater reliability from 0.63 to 0.88, and test-reliability was 0.90. This subscale was previously demonstrated to be moderately sensitive for the detection of anxiety in children with ASD^26^.

### The Autism Diagnostic Observation Schedule 2 (ADOS-2)^33^

ASD severity was examined using the ADOS-2, a semi-structured behavioral observation protocol that assesses ASD symptomatology. We used the Calibrate Severity Score (CSS), ranging from 1 to 10, a higher score corresponding to greater ASD severity. The internal consistency ranged from poor to excellent (α = 0.50–0.92), test–retest reliability was acceptable (0.64–0.88), and interrater reliability ranged from good to excellent (0.79–0.98).

### The Autism Diagnostic Interview-Revised (ADI-R)^34^

The ADI-R is a semi-structured interview that assesses two subdomains of ASD symptomatology Social Interaction (SI) and RRB, each items is scored from 0 (absence) to 3 (presence, severe impact). The range of scoring is 0–42 for the SI subdomain and 0–18 for the RRB subdomain.

The best-estimate IQ was assessed from psychometric scales, depending on the age of the participant^37^. The non-verbal cognitive level was estimated from the “fluid reasoning” dimension of the Wechsler Intelligence Scales for children WISC-V^38^ and WPPSI-IV^39^, the “perceptual reasoning” dimension of the WISC-IV^40^, and the “performance IQ” of the WPPSI-III^41^ or “simultaneous process” of the Kaufman Assessment Battery second version (K-ABC-II)^42^.

### The Vineland Adaptive Behavior Scales, Second Edition (VABS-II)^43^

Adaptive functioning was assessed using the VABS-II. This standardized caregiver interview of 297 items measures adaptive behaviors in the subdomains of communication, daily living skills, and socialization. Standard scores for VABS domains ranged from 20 to 160, with a mean of 100 and a standard deviation of 15; higher scores are indicative of better adaptive functioning. The reliability of the VABS-II for each domain was excellent (α = 0.80) and the intra-class coefficient of the test/re-test 0.89.

### The Aberrant Behaviors Checklist (ABC)^44^

Maladaptive behaviors were assessed using the ABC, a 58-item scale concerning problem behaviors, with each item scored from 0 (no problem) to 3 (severe). The scale includes four factors: (I) irritability, agitation, crying; (II) lethargy, social withdrawal; (III) stereotypic behavior; and (IV) hyperactivity, noncompliance. Scores were reduced to a scale of 100 to allow comparison. The ABC showed good internal consistency (α = 0.91), excellent test–retest reliability of 0.98, and acceptable interrater reliability of 0.63.

### The sensory profile questionnaire^45^

Sensory processing was assessed using the Sensory Profile questionnaire. A total score of the Sensory Profile can be calculated from 38 items extracted from the long version. Parents rated each behavior on a 5-point Likert scale to assess its frequency (1 = frequently occurring, 5 = rarely occurring). Lower scores indicate greater sensory processing difficulties. Internal consistency ranges from 0.70 to 0.90 and internal validity correlations from 0.25 to 0.76^46^.

### The hospital anxiety and depression scale (HADS)^47^

Parental anxiety was assessed by the HADS. Mothers and fathers completed the 14-item self-questionnaire. The thresholds for the sub-scores were: 0 to 7, absence of anxiety (low); 8 to 10, suspected anxiety (moderate); from 11 to 21, high level of anxiety (high).

### Parental socioeconomic status (SES)

SES is a composite variable based on the mother and/or father’s professional background. When the parents were living together, the highest SES in the household was considered; when separated, the SES of the parent with whom the child lived most of the time was considered. The SES was scored as high (business owners, professionals, executives), middle (farmers, supervisors, skilled craftsmen), or low (farm workers, laborers, service employees, unemployed).

Caregivers completed questionnaires electronically on a web database, including the Child Behavior Checklist (CBCL), the Sensory Profile, the Aberrant Behavior Checklist (ABC), the Hospital Anxiety and Depression Scale (HADS), and their socioeconomic status (SES).

### Statistical analysis

Descriptive and frequency statistical analyses were performed to characterize the sample according to the CBCL, socio-demographic data, and clinical variables at T1 and T2. The CBCL anxiety score at T2 was used to define children based on the level of anxiety according to the cut-off for the borderline clinical range: ASD-only group (CBCL score < 65) and ASD + anxiety group (CBCL score > 65). To identify early risk factors related to anxiety disorder, intergroup comparisons were performed using T1 clinical characteristics based on gender, chronological age, IQ, autism severity, and VABS-II, sensory profile, HAD, and ABC scores were conducted using X^2^ test, Student *t* tests for parametric variables and Mann–Whitney tests for non-parametric variables. The normality of the distributions was assessed by the Shapiro–Wilk Test. Two Effect Size (ES) measures were calculated: Cohen’s d (for *t* test or Mann–Whitney test) and Cohen’s w (for X^2^ test). Interpretation of effect sizes was based on guidelines presented by Cohen^48^ (small d ≤ 0.2, medium d = 0.5, and large d ≥ 0.8; small w ≤ 0.1, medium w = 0.3 and large w ≥ 0.5). Finally, multivariable logistic regression models were generated using the dependent variables (ADI-R, RRB score, sensory profile score, and ADOS-CSS score) significantly associated with the level of anxiety in the univariate analysis. Anxiety level measured with the CBCL and IQ both collected at baseline (T1) were entered in the model for adjustment in order to control potential confounding factors. The adjusted odds ratios (OR) and 95% confidence intervals (CI) are reported. We used backward selection to determine which variables to include in our model. The clinical characteristics of participants with missing outcome data did not differ from those with complete data. However, multiple imputations via fully conditional specification were used for the sensitivity multivariable logistic regression analysis. Adjusted odds ratios (aORs) were generated, accounting for the a priori selected confounding factors. The goodness-of-fit of the models was assessed using the Hosmer and Lemeshow chi-square test. Results were considered to be statistically significant for p < 0.05. Analyses were performed using SAS^®^ software v9.3.
